# Recovery Courses of Patients Who Return to Work by 3, 6 or 12 Months After Total Knee Arthroplasty

**DOI:** 10.1007/s10926-021-09959-6

**Published:** 2021-01-30

**Authors:** T. H. Hylkema, M. Stevens, J. van Beveren, P. C. Rijk, R. W. Brouwer, S. K. Bulstra, P. P. F. M. Kuijer, S. Brouwer

**Affiliations:** 1 Department of Orthopedics, University Medical Center Groningen, University of Groningen, PO Box 30.001, 9700 RB Groningen, The Netherlands; 2 Department of Community and Occupational Medicine, University Medical Center Groningen, University of Groningen, Groningen, The Netherlands; 3 Department of Orthopedics, Röpcke-Zweers Hospital Hardenberg, Hardenberg, The Netherlands; 4 Department of Orthopedics, Medical Center Leeuwarden, Leeuwarden, The Netherlands; 5 Department of Orthopedics, Martini Hospital Groningen, Groningen, The Netherlands; 6 Coronel Institute of Occupational Health, Amsterdam UMC, University of Amsterdam, Amsterdam Public Health Research Institute, Amsterdam, The Netherlands

**Keywords:** Knee replacement, Return to work, Recovery, Longitudinal, Outcomes

## Abstract

*Purpose* This study compared the preoperative levels and postoperative recovery courses of physical and mental impairments, activity limitations and participation restrictions of working-age patients who return to work (RTW) by 3, 6 or 12 months after total knee arthroplasty (TKA). *Methods* A prospective survey study including TKA patients (aged < 65) (n = 146) who returned to work (RdTW) in the first postoperative year. Three groups were compared: those who returned by 3 (n = 35), 6 (n = 40) or 12 (n = 29) months. Surveys were completed preoperatively and at 6 weeks and 3, 6 and 12 months postoperatively. Outcomes represented domains of the International Classification of Functioning, i.e. physical impairments (pain, stiffness, vitality), mental impairments (mental health and depressive symptoms), activity limitations (physical functioning) and participation restrictions (social and work functioning). *Results* Preoperative knee-specific pain and physical functioning levels were better among patients who RdTW by 3 months, compared to those who returned by 12 months. Patients who RdTW by 3 months experienced significantly better recovery from physical impairments than those who returned by 6 months (on general pain) or 12 months (on general and knee-specific pain and on stiffness). Patients returning by 3 months experienced significantly better recovery from activity limitations (on knee-specific physical functioning). *Conclusions* To optimize return to work outcome after TKA surgery, the focus should lie on physical impairments (general and knee-specific pain, stiffness) and activity limitations (knee-specific physical functioning) during recovery.

## Introduction

Total knee arthroplasty (TKA) is considered to be an effective surgical treatment for end-stage knee osteoarthritis [1]. The number of TKA procedures is expected to increase in the coming decades because of ageing populations [1], higher numbers of persons with obesity [2, 3], improved longevity of arthroplasties [4] and more widespread engagement in an active lifestyle [2]. The greatest increase in TKA is seen in patients aged 45–65, to further climb in future projections, while it is anticipated that by 2030 at least 60% of TKAs will be performed in the working-age population [5–7]. This trend coincides with delayed retirement, raising the number of working-age patients undergoing TKA surgery even further. For working TKA patients it is crucial to return to work (RTW) after surgery. While work is beneficial for physical and mental health, it is also important for socioeconomic reasons [8].

Several studies have examined RTW rates after TKA surgery and showed that most people return within the first year following surgery [9–11]. However, RTW timing in the first postoperative year varies greatly. For example, in the Netherlands guidelines prescribe steering patients back to the workplace by 3 months after surgery [12], but only 34% have returned by then [10]. By 6 months patients are considered as performing most activities of daily living again [13], but only 60% have returned to work (RdTW) at that point [10]. By 12 months, when patients are considered as having recovered [13, 14], 67% have RdTW [10, 15]. Similar or slightly better percentages are found in other Western countries like Canada and the United Kingdom [9, 16]. Hence when exactly people RTW in the first postoperative year varies greatly and might be considered suboptimal.

When people RTW after surgery, they are recovering or feel they have recovered from the surgical intervention. It can be hypothesized that patients who RTW early or later postoperatively experience different recovery courses. So far, literature has mainly investigated preoperative predictors for RTW [9, 15, 17–19]. Only one study describes postoperative knee-specific pain and function levels at the time patients actually RTW [16], showing that those who return by 3 months instead of 6 or 12 months experience less pain and better function [16]. However, it can be presumed that time to RTW is explained not only by recovery outcomes such as knee-specific pain and function: postoperative work limitations—including physical (kneeling, bending) and mental limitations (fatigue, limited concentration)—are also factors found to be associated with later RTW [16]. Qualitative studies show that psychological and social factors also influence time to RTW [20, 21].

To obtain a complete picture of recovery, thereby explaining why some TKA patients may RTW later after surgery, it would be valuable to use a biopsychosocial approach to recovery that includes measures on physical and mental impairments, activity limitations and participation restrictions [22]. In addition, measuring recovery at multiple postoperative follow-up moments can help display the development of recovery and subsequently detect distinct courses of recovery for patients who RTW by 3, 6 or 12 months after surgery. Insight into these recovery courses of patients with varying RTW might help optimize rehabilitation care interventions and steer patients back to work earlier. The present study therefore aimed to compare the preoperative levels and postoperative recovery courses of physical and mental impairments, activity limitations and participation restrictions of TKA patients who RdTW by 3, 6 or 12 months.

## Methods

### Study Design and Participants

This prospective cohort study, titled “Work participation in Patients with Osteoarthritis” (WIPO), included consecutive patients with knee osteoarthritis scheduled for TKA between March 2012 and July 2014 in the northern Netherlands. Preoperative and postoperative data at 6 weeks and at 3, 6, 12 and 24 months were gathered by filling in a questionnaire. Inclusion criteria were primary or secondary knee OA and undergoing TKA, preoperative employment, and age 18–63. The age of 63 was chosen in order to complete the two-year follow-up before the (former) Dutch retirement age of 65. Exclusion criteria were insufficient knowledge of the Dutch language and having undergone joint arthroplasty in the previous 6 months. Patients were approached from waiting lists of the participating hospitals. Four hospitals participated: University Medical Center Groningen (UMCG; tertiary university hospital), Medical Center Leeuwarden (MCL; large teaching hospital), Martini Hospital Groningen (MHG; large teaching hospital) and Röpcke-Zweers Hospital Hardenberg (general hospital). The study was approved by the Medical Ethical Committee of University Medical Center Groningen (METc 2012.153) and is in accordance with the 1964 Helsinki declaration and its later amendments or comparable ethical standards. Informed consent was considered as obtained if the subject granted our request to participate in the study by returning a set of completed questionnaires. Subjects who did not want to participate were requested to return a blank questionnaire. Subjects were informed of this consent procedure by mail. In this letter they were also informed of the voluntary nature of the study, and that all data would be processed anonymously. The Medical Ethical Committee approved this consent procedure.

### Return to Work

RTW was measured based on response to the question “Which of the following responses describes your current employment status?”. Possible answers included “no, still on sick leave”, “yes, partial RTW” and “yes, full RTW”. Those who answered full RTW were considered as having returned at the first reporting moment. Time was coded as the first follow-up moment at which the participant indicated having first RTW, correspondingly resulting in three groups to be analyzed—RTW by 3, 6 or 12 months.

### Measures

Outcomes were grouped following the World Health Organization framework of International Classification of Functioning, Disability and Health (ICF) [22], considering physical and mental impairments, activity limitations and participation restrictions. The selection of outcome measures and classification in the ICF model was guided by a previous study that examined the discriminant validity of commonly used TKA outcome measures with ICF constructs [23].

#### Physical and Mental Impairments

Impairments were divided into physical impairments and mental impairments. *Physical impairments* included general and knee-specific pain, vitality and stiffness. General pain and vitality were measured with the equivalently named subscales of the Dutch version of the RAND 36-item Health Survey, which measures health-related quality of life [24]. General pain includes two questions on severity of pain and vitality includes four questions on energy level and fatigue. Subscore range is 0–100 and higher scores reflect less pain and better vitality. The RAND-36 is a reliable and valid instrument [24]. Knee-specific pain and stiffness were measured by the Dutch version of the Western Ontario and McMaster Osteoarthritis Index (WOMAC), transforming these subscales to a 0–100 scale. A higher score represents less pain or stiffness. The WOMAC has proven to be valid and reliable [25, 26].

The *mental impairments* construct included mental health and depressive symptoms. Mental health was captured by the subscale of the RAND-36 and asks about depressive symptoms and anxiety using five questions. Subscore range is 0–100 and higher scores reflect better mental health [24]. Depressive symptoms were measured by the PHQ-9 questionnaire, the depression module from the PRIME-MD instrument for common mental disorders, which scores each of the 9 DSM IV criteria from 0 (“not at all”) to 3 (“nearly every day”). Participants were asked how often they have been bothered by each of the depressive symptoms over the last 2 weeks. The PHQ-9 is a valid and reliable instrument [27]. The scale was recoded to a 0–100 scale, with higher scores representing fewer depressive symptoms.

#### Activity Limitations

The *activity limitations* construct was captured by self-reported general and disease-specific physical functioning, measured by subscales of both the RAND-36 [24] and the WOMAC [26], respectively. Physical functioning of the RAND-36 asks about restrictions in performing ten daily activities due to health problems, such as climbing stairs, doing laundry and household chores. Range is 0–100 and higher scores reflect better physical functioning. Knee-specific physical functioning was measured by the WOMAC and covers limitations in physical functioning because of knee complaints during daily activities (17 items). A higher score (range 0–100) indicates better knee-specific physical functioning.

#### Participation Restrictions

The *participation restriction* construct was measured by including social and work functioning. Social functioning was measured by the RAND 36-item social functioning subscale and asks about restrictions due to health problems in social activities such as visiting friends. Subscore ranges are 0–100 and higher scores reflect better social functioning [24]. Work functioning was measured by the Work Role Functioning Questionnaire 2.0 (WRFQ), which measures self-reported difficulties in meeting 27 work demands among workers, given their physical health or emotional problems. The total score was used, which ranges from 0 to 100, with higher scores indicating better work functioning. The WRFQ was not assessed at 6 weeks postoperatively due to a high possibility that many patients would not be at work. The WRFQ 2.0 has good internal consistency, moderate test–retest reliability and moderate responsiveness in the general working population [28].

### Sociodemographic Variables

The preoperative questionnaire included questions on age, gender, educational level, home situation, body mass index (BMI), comorbidity, being the wage earner (yes/no), type of employment, and job type. Educational level was categorized into lower (primary school and lower vocational education), intermediate (secondary and regular vocational education) and higher (higher vocational education and university). Home situation was categorized as living alone, living with partner or living with partner and children. BMI was derived from self-reported body height and body weight, and categorized into normal (18.5–24.9 kg/m^2^), overweight (25.0–29.9 kg/m^2^), and overweight/obese (> 30.0 kg/m^2^). Comorbidity was measured by using a 27-item chronic conditions questionnaire from Statistics Netherlands [29]. Number of comorbidities was categorized into having no, one or two, or more comorbidities. Employment type included being self-employed or salaried. Job type inquired about type of work with three possible answers: mainly physically demanding tasks, mainly mentally demanding tasks, or a combination of the two.

### Statistical Analyses

All analyses were conducted using SPSS version 23. First, patients’ sociodemographic characteristics were analyzed using descriptive statistics (mean, median or percentage) and tested between the groups by one-way ANOVA (continuous coded covariates) or Chi square tests (categorical coded covariates). Next, preoperative levels and recovery courses of physical and mental impairments, activity limitations and participation restrictions during a 12-month follow-up were analyzed by RTW status. Given that most patients RdTW within the first 12 months, 24-month data could not be used as groups were too small to be analyzed. Linear mixed models (LMM) were used to calculate the estimated means with corresponding 95% confidence intervals (CIs). RTW status was added as an interaction term to the analyses. Preoperative differences between the groups were analyzed using the model. Differences between the recovery courses of all groups were also analyzed, as were differences between the recovery courses of two groups, i.e. those who returned by 3 months versus 6 or 12 months and those who returned by 6 months versus 12 months. Differences on individual follow-up moments were likewise reported. All models were adjusted for age, gender and comorbidity. Results were tabulated and overall significant different courses were graphed. Lastly, an attrition bias analysis was conducted comparing non-responders’ age, gender, BMI, comorbidity, educational level and home situation at 24 months with responders. This analysis revealed no significant differences. Statistical significance was set at p < 0.05.

## Results

A total of 180 TKA patients were eligible and invited to participate. At baseline, n = 146 (81%) returned the questionnaire and were included. Analyses were conducted on n = 104 patients who reported having RdTW in the course of the 12-month follow-up. By 3 months, n = 35 (24.0%) had returned, by 6 months n = 40 (27.3%, cumulative 51%), and by 12 months n = 29 (19.8%, cumulative 71%). Four (2.7%) patients reported not having RdTW 12 months post-surgery and 10 patients (6.8%) had returned partially. The other patients (28) were lost to follow-up. Baseline characteristics of the three groups are presented in Table 1. No significant differences between groups were found for the included sociodemographic variables of age, gender, BMI, comorbidity, educational level, home situation, being the main breadwinner, type of employment or job type.

**Table 1:** Baseline characteristics

Sociodemographic variables	RTW by 3 mo (n = 35)	RTW by 6 mo (n = 40)	RTW by 12 mo (n = 29)	p value
Age (mean (SD) (range))	55.1 (6.1) (38-62)	55.8 (4.1) (45-62)	54.3 (5.4) (41-62)	0.55
Female gender (no (%))	18 (51)	24 (60)	18 (62)	0.57
BMI (no (%))				0.54
18.5-24.9	5 (14)	9 (23)	3 (10)	
25.0-29.9	18 (51)	17 (43)	11 (38)	
> 30.0	11 (22)	14 (35)	13 (45)	
Comorbidity (no (%))				0.42
0	4 (11)	2 (5)	1 (3)	
1	5 (14)	11 (28)	7 (24)	
2 or more	24 (69)	26 (65)	12 (41)	
Educational level (no (%))				0.74
Lower	13 (37)	14 (35)	8 (28)	
Intermediate	13 (37)	20 (50)	13 (45)	
Higher	7 (20)	5 (12)	6 (21)	
Home situation (no (%))				0.43
Living alone	4 (11)	1 (3)	4 (14)	
Living with partner	22 (62)	23 (58)	14 (48)	
Living with partner and children	9 (26)	15 (38)	10 (34)	
Being the wage earner (no (%))	20 (57)	18 (45)	15 (52)	0.56
Type of employment (no (%))				0.07
Self-employed	9 (26)	4 (10)	2 (6)	
Salaried	26 (74)	36 (90)	26 (90)	
Job type (no (%))				0.62
Primarily physically demanding tasks	10 (29)	13 (33)	10 (34)	
Primarily mentally demanding tasks	15 (43)	11 (28)	7 (24)	
Both mentally and physically demanding tasks	10 (29)	15 (38)	10 (34)	

*N* may vary due to missing data, *mo* months, *BMI* body mass index

### Physical and Mental Impairments

**Fig. 1:**
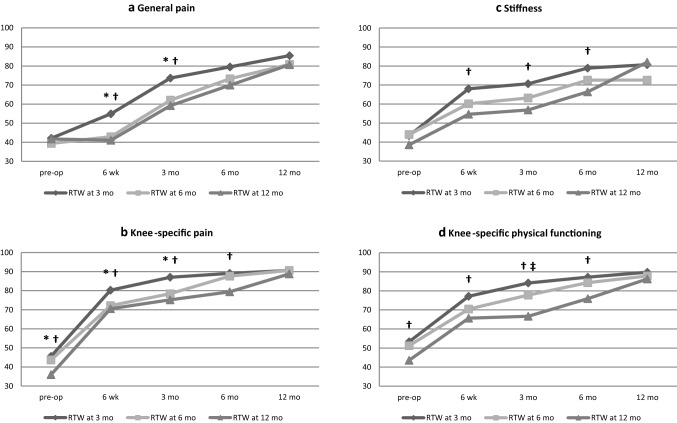
**a**, **b**, **c**, **d** Recovery courses of general and knee-specific pain, stiffness and knee-specific physical functioning between those who RTW by 3, 6 or 12 months (all p < 0.05). *mo* months. *Significant difference between RTW at 3 months and at 6 months. ^†^significant difference between RTW at 3 months and at 12 months. ^‡^Significant difference between RTW at 6 months and at 12 months

Preoperative knee-specific pain levels were significantly lower among patients who RdTW by 3 months compared those who returned by 12 months. No significant preoperative differences on knee-specific pain were found between RTW by 3 and 6 months or between 6 and 12 months, or for general pain, stiffness, vitality or mental impairments (Table 2). Significantly different courses of recovery were found for general and knee-specific pain and for stiffness (Fig. 1, Table 2). No significantly different recovery courses of vitality or mental impairments were observed between the three groups.

**Table 2:** Recovery courses of physical and mental impairments, activity limitations and participation restrictions for those who returned to work by 3, 6 or 12 months

Construct	Measures (instrument)	RTW groups	Preoperatively	6 weeks	3 months	6 months	12 months	Overall p value^a^	*p* value 3-6mo^b^	p value 3-12mo^c^	p value 6-12mo^d^
			Estimate (95% CI)	Estimate (95% CI)	Estimate (95% CI)	Estimate (95% CI)	Estimate (95% CI)				
Physical Impairments	General pain (RAND-36)	RTW by 3 mo	42.5 (34.3–50.7)	55.3 (47.1–63.5)*†	74.0 (65.8–82.2)*†	79.8 (71.5–88.1)	85.8 (77.6–94.0)	**0.01**	**0.01**	**0.01**	0.74
		RTW by 6 mo	38.7 (31.3–46.1)	42.2 (34.6–49.8)*	61.5 (53.9–69.0)*	72.7 (65.3–80.0)	80.2 (72.7–87.7)				
		RTW by 12 mo	41.1 (31.6–50.5)	**40.4 (30.8–50.0)†**	**58.6 (49.1–68.0)†**	69.2 (59.6–78.8)	80.0 (70.6–89.5)				
	Knee-specific pain (WOMAC)	RTW by 3 mo	**46.2 (39.3–53.0)†**	80.9 (74.0–87.7)*†	87.7 (80.8–94.6)*†	**89.6 (82.7–96.6)†**	91.2 (84.2–98.2)	**0.02**	0.10	**0.005**	0.15
		RTW by 6 mo	43.4 (37.2–49.5)	71.9 (65.6–78.2)*	78.2 (71.8–84.5)*	87.3 (81.2–93.5)	90.3 (84.0–96.6)				
		RTW by 12 mo	**35.5 (27.6–43.4)†**	**70.1 (62.2–77.9)†**	**74.7 (66.6–82.8)†**	**78.9 (70.9–86.9)†**	88.3 (80.5–96.2)				
	Vitality (RAND-36)	RTW by 3 mo	60.2 (53.1–67.3)	63.7 (56.6–70.8)	**70.8 (63.7–77.9)†**	69.0 (61.8–76.2)	70.4 (63.3–77.6)	0.36	0.28	0.18	0.67
		RTW by 6 mo	55.8 (49.4–62.1)	58.5 (52.0–65.0)	63.1 (56.7–69.6)	70.5 (64.2–76.8)	69.5 (63.0–75.9)				
		RTW by 12 mo	58.9 (50.9–67.0)	55.2 (47.1–63.4)	**61.1 (53.0–69.2)†**	66.7 (58.5–74.9)	68.0 (59.9–76.1)				
	Stiffness (WOMAC)	RTW by 3 mo	42.3 (34.3–50.4)	**66.9 (58.9–75.0)†**	**69.6 (61.5–77.7)†**	**77.8 (69.6–85.9)†**	79.6 (71.5–87.)	**0.04**	0.07	**0.02**	0.43
		RTW by 6 mo	42.5 (35.2–49.7)	58.6 (51.3–66.0)	61.6 (54.2–69.1)	71.1 (63.8–78.3)	71.1 (63.7–78.4)				
		RTW by 12 mo	36.9 (27.6–46.2)	**53.0 (43.7–62.2)†**	**55.2 (45.8–64.6)†**	**64.7 (55.2–74.3)†**	80.3 (71.1–89.6)				
Mental impairments	Mental health (RAND-36)	RTW by 3 mo	77.8 (72.1–83.5)	81.6 (75.9–87.2)	86.2 (80.58–91.8)	84.1 (78.4–89.9)	83.1 (77.4–88.8)	0.71	0.41	0.69	0.74
		RTW by 6 mo	76.2 (71.1–81.2)	77.5 (72.3–82.6)	81.6 (76.5–86.7)	83.7 (78.6–88.7)	83.1 (78.0–88.2)				
		RTW by 12 mo	78.1 (71.1–84.5)	78.6 (72.2–85.1)	83.8 (77.4–90.2)	84.0 (77.5–90.4)	82.3 (75.9–88.7)				
	Depressive symptoms (PHQ-9)	RTW by 3 mo	85.0 (79.4–90.6)	82.3 (76.7–87.8)	87.5 (82.0–93.1)	90.8 (85.1–96.5)	90.7 (85.1–96.4)	0.51	0.35	0.29	0.80
		RTW by 6 mo	83.3 (78.2–88.5)	78.6 (73.5–83.8)	86.0 (81.0–91.0)	91.0 (86.0–96.0)	86.2 (81.1–91.2)				
		RTW by 12 mo	85.9 (79.4–92.5)	77.1 (70.7–83.5)	81.0 (74.7–87.4)	89.8 (83.4–96.2)	87.9 (81.6–94.3)				
Activity limitations	General physical functioning (RAND-36)	RTW by 3 mo	33.7 (25.8–41.5)	**49.5 (41.6–57.3)†**	**61.0 (53.1–68.8)†**	67.1 (59.1–75.0)	72.6 (64.8–80.5)	0.07	0.24	**0.02**	0.18
		RTW by 6 mo	29.2 (22.2–36.2)	40.8 (33.6–48.0)	55.6 (48.3–63.0)	66.2 (59.2–73.2)	72.5 (65.3–79.7)				
		RTW by 12 mo	27.4 (18.4–36.5)	**38.2 (29.1–47.4)†**	**47.8 (38.8–56.8)†**	59.9 (50.7–69.0)	66.9 (57.9–76.0)				
	Knee-specific physical functioning (WOMAC)	RTW by 3 mo	**51.7 (45.2–58.3)†**	**75.6 (69.0–82.1)†**	**82.6 (76.0–89.1)†**	**85.6 (78.9–92.2)†**	88.2 (81.6–94.8)	**0.005**	0.13	**0.001**	**0.04**
		RTW by 6 mo	49.3 (43.5–55.2)	68.6 (62.6–75.5)	**75.8 (69.8–81.8)‡**	82.4 (76.5–88.2)	85.9 (79.9–91.9)				
		RTW by 12 mo	**42.0 (34.5–49.5)†**	**64.1 (56.6–71.6)†**	**65.1 (57.6–72.6)†‡**	**74.3 (66.7–81.9)†**	84.7 (77.2–92.2)				
Participation restrictions	Social functioning (RAND-36)	RTW by 3 mo	73.6 (65.4–81.9)	72.1 (63.9–80.4)*	84.2 (76.0–92.5)	91.7 (83.3–100.1)	90.3 (82.0–98.6)	0.14	0.06	0.14	0.84
		RTW by 6 mo	65.4 (58.0–72.8)	60.9 (53.3–68.6)*	79.4 (71.8–87.1)	87.8 (80.4–95.2)	87.1 (79.5–94.7)				
		RTW by 12 mo	71.9 (62.3–81.4)	62.8 (53.1–72.5)	76.6 (67.1–86.2)	83.4 (73.7–93.1)	89.7 (80.2–99.3)				
	Work functioning (WRFQ)	RTW by 3 mo	78.2 (71.0–85.3)	n.a.	**89.2 (81.9–96.6)†**	87.9 (80.5–95.4)	94.3 (87.1–101.6)	0.16	0.60	0.06	0.11
		RTW by 6 mo	78.4 (71.9–85.0)	n.a.	83.1 (76.3–90.0)	88.6 (82.4–94.9)	92.6 (86.3–98.9)				
		RTW by 12 mo	82.2 (72.8–91.5)	n.a.	**68.2 (54.2–82.3)†**	81.3 (71.6–91.0)	85.1 (76.3–93.8)				

Bold values indicates statistical significance (p ≤ 0.05)

All models were adjusted for age, gender and comorbidity

*mo* months

^a^Overall p value between all groups

^b^Difference between recovery of those who RTW by 3 vs. by 6 months

^c^Difference between recovery of those who RTW by 3 vs. by 12 months

^d^Difference between recovery of those who RTW by 6 vs. by 12 months

^*^Significant difference (p ≤ 0.05) between those who RTW by 3 vs. by 6 months at a specific timepoint

^†^Significant difference (p ≤ 0.05) between those who RTW by 3 vs. by 12 months at a specific timepoint

^‡^Significant difference (p ≤ 0.05) between those who RTW by 6 vs. by 12 months at a specific timepoint

For general pain, patients who RdTW by 3 months compared to 6 or 12 months reported significantly better recovery from pain. The 6- and 12-month groups reported more pain on the 6-week and 3-month follow-up moments than the 3-month group. Patients who returned by 3 months improved directly after surgery, while the 6- and 12-month groups only started to improve at 6 weeks (Table 2, Fig. 1a).

In terms of knee-specific pain, patients who RdTW by 3 months were experiencing better recovery courses than those returning by 12 months. No significant differences between the other groups were found. Knee-specific pain levels were lower for the 3-month group at the 6-week, 3-month and 6-month follow-up moments than for the other groups. All groups improved after surgery, but those who returned by 12 months instead of 3 months had a slower decrease in pain (Table 2, Fig. 1b).

Patients who RdTW by 3 months instead of 12 months reported significantly better recovery from stiffness. Stiffness was lower for the 3-month group at 6 weeks and at 3 and 6 months than for the 12-month group. All groups followed the same course of recovery, though those who returned by 3 months reported greater improvements in the first 6 weeks postoperatively (Fig. 1c).

### Activity Limitations

Preoperative knee-specific physical functioning levels were significantly lower for those who RdTW by 3 months than for those returning by 12 months. No significant preoperative differences were found between the other groups or for general physical functioning (Table 2).

Recovery of knee-specific physical functioning was significantly better among patients who RdTW by 3 months compared to the 12-month group. Further, those who returned by 6 months experienced better recovery of knee-specific physical functioning compared to the 12-month group. Specifically at 6 weeks, 3 months and 6 months, the 3-month group scored better than the 12-month group. At the 3-month follow-up moment, the 6-month group scored better than the 12-month group (Table 2, Fig. 1). All groups showed improvements directly after surgery, but those who returned by 12 months improved in a slower pattern and showed no significant improvement specifically between 6 weeks and 3 months (Table 2, Fig. 1d).

Recovery courses of general physical functioning were not significantly different overall (p = 0.07), but patients who RdTW by 3 months instead of 12 months experienced significantly better recovery of general physical functioning. The groups returning by 3 and 12 months specifically differed at the 6-week and 3-month follow-up moments (Table 2).

### Participation Restrictions

No significant differences were observed in preoperative levels of either social or work functioning, or for the recovery courses of both participation outcomes between the three groups. Only at two specific follow-up moments were significantly different levels observed. Six weeks postoperatively, patients who RdTW by 3 months experienced better social functioning compared to those returning by 6 months. Three months postoperatively, those who returned by 3 months experienced better work functioning than those returning by 12 months (Table 2).

## Discussion

This study compared working TKA patients who RdTW by 3, 6 or 12 months postoperatively on both preoperative status and recovery courses. No significant differences were found preoperatively on sociodemographic characteristics between these groups. However, patients who returned by 3 months experienced less knee-specific pain and better physical functioning preoperatively than those returning by 12 months. Patients who returned by 3 months compared to those returning by 6 or 12 months reported better recovery courses from physical impairments in terms of general and knee-specific pain as well as stiffness. No significant differences in recovery from mental impairments were observed. Recovery courses from activity limitations with respect to knee-specific physical functioning were better for those returning by 3 months compared to 12 months, and for 6 months compared to 12 months. No significant different recovery courses from participation restrictions were observed between the three groups.

To our knowledge, this is the first prospective study to examine and compare recovery courses for TKA patients who RdTW by 3, 6 or 12 months by collecting data at multiple time points postoperatively. Of course, several findings need discussion and comparison with literature. At first, the observed RTW rates at 6 and 12 months were in line with literature, namely 51% and 71%, but the 24% RTW rate at 3 months was lower than that found in other studies [10, 16]. An explanation might be that we chose to define RTW as having returned to work full-time, while others only asked about RTW status (yes/no) or also included participants who had partially returned. In the present study, for example when a person had partially returned at 3 months, they would only be included at 6 months if they had returned to full employment by then. Moreover, RTW rates cannot be directly compared to other countries due to differences in healthcare funding and insurance systems and the linkage between employment and different welfare and worker compensation schemes [9].

Preoperative differences in knee-specific pain and function were found between those who RdTW by 3 or by 12 months, without any differences in sociodemographic characteristics between these two groups. Previous RTW literature on TKA patients mainly focused on preoperative predictors for RTW (yes/no). Only one study prospectively examined time taken to RTW [17], and found several sociodemographic characteristics associated with faster or slower RTW, like being female, being self-employed and having a physically demanding job [17]. Those results are in contrast to the present study, as we found no significant preoperative differences in sociodemographic characteristics between the three groups. Better preoperative physical function scores were associated with faster RTW [17], which is in line with the present study, but in contrast to our results that study found that having less pain preoperatively was associated with slower RTW. Clinically it seems more logical for less pain to be associated with earlier RTW, as the present study found.

Patients were mainly hampered by knee-related symptoms like pain and stiffness, as well as by general pain which might originate from body regions other than the operated knee. Comparable differences in postoperative knee-specific pain levels were observed earlier by Sankar et al., with participants who RdTW between 6 and 12 months instead of 3 months reporting higher pain levels at the time of return [16]. The differences in recovery courses of general and knee-specific pain are of interest. Knee-specific pain levels improved directly after surgery, albeit at a different pace in every group. General pain followed a different course, whereby patients who RdTW by 6 or 12 months experienced no improvements in general pain in the first 6 weeks postoperatively and subsequently followed similar courses. General pain asks about pain in the body without specifically inquiring about pain from the operated knee [24]. Patients who RTW after more than 3 months might therefore be hampered by comorbidities other than the operated knee or, for example, by pain behavior like pain catastrophizing. No significant preoperative differences in prevalence of comorbidity were found. However, prevalence does not represent disease severity and therefore might have biased this outcome. On the other hand, pain catastrophizing could also have an effect, as it has been associated with absenteeism among TKA patients [30]. Lastly, stiffness hindered patients from returning to work—a novel finding, as most RTW studies only include pain and function. Stiffness is a common symptom after TKA surgery [31] and is associated with severe postoperative dissatisfaction among working-age patients [32].

We found no significant differences in recovery from mental impairments between the three groups. By contrast, Styron et al. found that better preoperative mental health status was associated with earlier RTW [17]. Differences might be explained by the fact that our patients were not experiencing any more mental health problems or depressive symptoms preoperatively than the general working population based on normative values [24, 27]. Small improvements were made after surgery, mainly on depressive symptoms, which can be attributed to pain relief [27].

For activity limitations, patients returning to work by 3 months showed improvements on knee-specific physical functioning directly after surgery, while those returning by 6 months and 12 months improved in a slower pattern. These results are in line with Sankar et al., who also described better functional levels for participants returning by 3 months than those returning later [16]. When comparing the physical functioning levels at the time our three groups RdTW, levels of at least 82.0% for knee-specific physical functioning were reached at the time of return. For general physical functioning a minimum of 61.0% was measured at the time of return. This might suggest that TKA patients do need to experience a minimal level of functioning before being able to RTW.

Recovery courses of participation restrictions, including social and work functioning, did not differ over a 12-month period between groups. Only significant differences at two single follow-up moments were found for both outcomes. Social functioning level differed at 6 weeks postoperatively between those who RdTW by 3 months instead of 6 months. We could not explain this finding, as the 12-month group did not have a different score than the 3- and 6-month groups. Work functioning improved in the first 6 weeks postoperatively among persons who returned by 3 or 6 months instead of 12 months. Patients who returned by 12 months reported lower work functioning at 3 months postoperatively, compared to their preoperative state (mean score: 68.2 versus 82.2). Work functioning is considered as “good” at 90 points or higher, which represents having the ability to meet the demands of work for a given state of health [28]. From that perspective, those who returned by 12 months were not yet experiencing good work functioning at the time of return (85.1), while work functioning levels of the 3- and 6-month groups exceeded the 90 points at 12 months postoperatively.

### Strengths and Limitations

A strength of this study is that we reported from a biopsychosocial perspective, which captured more than solely knee-specific pain or functioning. Another strength is the heterogeneous sample of patients, as they were derived from primary, secondary and tertiary hospitals. This improves the generalizability of our results. We are aware that the knee-specific pain subscale of the WOMAC represents both physical impairments and activity limitations [23]. We nonetheless decided to include knee-specific pain as physical impairment and not leave it out, in order to compare with literature and deepen insights into pain experiences. An additional limitation was the sample size, impeding full adjustment for covariates other than age, gender and comorbidity. Another limitation is the lack of continuous data on RTW status, as those who answered having RdTW at the 3-, 6- or 12-month point could have returned to work earlier.

### Implications and Recommendations

The present study shows that working patients who RdTW later than 3 months after surgery are mainly hampered in their recovery by general and knee-specific pain, stiffness, and knee-specific physical functioning. Delayed RTW may therefore be ascribed to problems related to the TKA, but ongoing pain from body regions other than the operated knee may also be relevant. For clinical practice, several preoperative and postoperative recommendations might support these TKA patients to RTW earlier. Preoperatively, worse knee-specific pain and physical functioning might be useful to screen those at risk of delayed RTW. It is worth considering operating on these patients at an earlier stage, when pain levels are lower and RTW outcome might be optimized. Postoperatively it is known that support from physical therapists and orthopedic surgeons helps improve clinical outcomes in TKA patients [33] and may facilitate RTW of sick-listed workers [34]. However, a recent review concluded that such effective work-directed and tailored rehabilitation programs are not available yet [35]. Future studies are warranted to evaluate whether tailored and work-directed rehabilitation is possible with proper timing and provision of rehabilitative support [35]. Finally, it is of interest to further investigate sustainable RTW after TKA, where changes in RTW status (i.e. changing from having RdTW at 3 months to sick leave at 12 months) are more thoroughly analyzed in prospective studies.

## Conclusion

This prospective study showed that TKA patients who RTW at 3, 6 or 12 months postoperatively differ preoperatively on knee-specific pain and physical functioning but not on sociodemographic characteristics. Patients who RTW by 3 months experience better recovery from physical impairments than those who return by 6 months on general pain and better recovery from general and knee-specific pain and stiffness compared those who return by 12 months. Recovery of knee-specific physical functioning was better among those who returned by 3 months compared 12 months, and better among those who returned by 6 months compared to 12 months. Patients experiencing delayed RTW are therefore hampered mainly by knee-related problems, which highlights where to support these patients in clinical practice.
